# Enrichment, Development, and Assessment of Indian Basil Oil Based Antiseptic Cream Formulation Utilizing Hydrophilic-Lipophilic Balance Approach

**DOI:** 10.1155/2013/410686

**Published:** 2013-07-31

**Authors:** Narayan Prasad Yadav, Jaya Gopal Meher, Neelam Pandey, Suaib Luqman, Kuldeep Singh Yadav, Debabrata Chanda

**Affiliations:** ^1^Herbal Medicinal Products Department, CSIR-Central Institute of Medicinal and Aromatic Plants, PO-CIMAP, Lucknow 226015, India; ^2^Molecular Bioprospection Department, CSIR-Central Institute of Medicinal and Aromatic Plants, PO-CIMAP, Lucknow 226015, India

## Abstract

The present work was aimed to develop an antiseptic cream formulation of Indian basil oil utilizing hydrophilic-lipophilic balance approach. In order to determine the required-hydrophilic lipophilic balance (rHLB) of basil oil, emulsions of basil oil were prepared by phase inversion temperature technique using water, Tween 80, and Span 80. Formulated emulsions were assessed for creaming (BE9; 9.8, BE10; 10.2), droplet size (BE18; 3.22 ± 0.09 **μ**m), and turbidity (BE18; 86.12 ± 2.1%). To ensure correctness of the applied methodology, rHLB of light liquid paraffin was also determined. After rHLB determination, basil oil creams were prepared with two different combinations of surfactants, namely, GMS : Tween 80 (1 : 3.45) and SLS : GMS (1 : 3.68), and evaluated for *in vitro* antimicrobial activity, skin irritation test, viscosity and consistency. The rHLB of basil oil and light liquid paraffin were found to be 13.36 ± 0.36 and 11.5 ± 0.35, respectively. Viscosity, and consistency parameters of cream was found to be consistent over 90 days. Cream formulations showed net zone of growth inhibition in the range of 5.0–11.3 mm against bacteria and 4.3–7.6 mm against fungi. Primary irritation index was found to be between 0.38 and1.05. Conclusively stable, consistent, non-irritant, enriched antiseptic basil oil cream formulations were developed utilizing HLB approach.

## 1. Introduction

*Ocimum basilicum* L. (family Lamiaceae), popularly known as Indian basil or sweet basil, is an illustrious plant used as kitchen herb in culinary and as an ornamental plant in traditional medicine. The anti-inflammatory [1], antimicrobial [2], insecticidal [3], hypolipidemic [4], antioxidant, and radical scavenging [5] activities of *Ocimum* have been ascertained in a number of published reports, and basil has been integrated in a wide variety of herbal preparations for the treatment of various ailments [6, 7]. Little or no information is available about any potential toxicity of basil oil in humans. No health hazards or side effects are known in conjunction with the proper administration of designated therapeutic dosages. But due to the high estragole content, basil oil/the herb should not be taken during pregnancy [8, 9]. No reports are available about any possible side effects on external usage of basil oil. It is a light yellow color liquid with characteristic odor, insoluble in water and soluble in vegetable oils having flash point (176°C) and specific gravity (0.905–0.955 g/cm^3^), and reported to possess potent broad spectrum antimicrobial activity [8–10].

Hydrophilic-lipophilic balance (HLB) value is not only an important parametric scale for the selection of surfactants but also a critical step for the development of emulsion formulation and can be employed as an utmost factor for quality control [11–13]. Hence, rHLB of each ingredient in an emulsion must be known before proceeding towards formulation and development. Till now, rHLB of very few essential oils has been reported, and it is one of the important factors to develop stable emulsion [14].

Our present investigation was aimed to determine the rHLB of basil oil employing Stokes' law and to utilize the same in the development of antiseptic cream formulation. Droplet size analysis, creaming index, and turbidity of emulsion were selected as critical parameters in the determination of rHLB. Light liquid paraffin was chosen as a reference standard (HLB 11 ± 1) to ensure correctness of the applied methodology [15]. Implementing the determined rHLB of basil oil, elegant cream formulations were developed and evaluated for consistency, viscosity, antimicrobial action, and safety for topical application.

## 2. Materials and Methods

### 2.1. Materials

Basil oil was obtained from CSIR-Central Institute of Medicinal and Aromatic Plants (CSIR-CIMAP), Lucknow, India. Light liquid paraffin, Tween 80, Span 80, stearyl alcohol, cetyl alcohol, hard paraffin wax, sodium lauryl sulfate (SLS), glycerol monostearate (GMS), white petroleum jelly, and sodium chloride were procured from SD Fine Chem Limited, Mumbai. Nutrient agar, sabouraud dextrose agar, agar powder, and disposable petri dishes were purchased from HiMedia Laboratories Pvt. Ltd., Mumbai, India. All ingredients were of analytical grade and used as received. A popular (Indian) herbal antiseptic cream was procured from local market and used as reference standard for antimicrobial evaluation and skin irritation test.

### 2.2. Methods

#### 2.2.1. Preparation of Emulsion

Phase inversion temperature technique was employed for the preparation of emulsions [16, 17]. For this purpose, 5% of basil oil/light liquid paraffin was taken in oil phase, blends of Tween 80 (HLB 15.0) and Span 80 (HLB 4.3) in different ratio were chosen as emulsifier at 3% concentration, and water (quantity sufficient) was taken in the aqueous phase. Emulsifier blends which could produce HLB in the range of 5–15 were prepared separately for both basil oil and light liquid paraffin in first series of preparations whereas in the second series, eight emulsions in HLB range of 11.0–14.0 were formulated. Hydrophilic-lipophilic balance spreadsheet for emulsions is depicted in Table 1.

#### 2.2.2. Evaluation of Emulsions****

*Creaming Index (CI)*. CI was determined by taking the ratio of total height of cream (*H*_*C*_) layer and total height of emulsion (*H*_*E*_) in the storage container (plastic bottle with 3 cm diameter and 10 cm height). CI was determined on 5th, 10th, 30th, 45th, and 60th day using the following formula [18]:

(1) $$\begin{array}{c} \text{CI}\%=\left(\frac{H_{C}}{H_{E}}\right)\times 100. \end{array}$$

*Turbidity Evaluation.* Turbidity evaluation of the formulated emulsions was performed as per previously reported method [18]. Briefly, the sample was diluted to 25 mL with double distilled water, and the percent transmission (*T*%) was measured at 600 nm with the help of UV-Visible Spectrophotometer (SpectraMax, Molecular Devices, USA) keeping blank control at 100% transmission. Turbidity of the emulsions was determined on 7th and 60th day of storage. Sampling was done in triplicate, and the mean value was calculated.

*Droplet Size Evaluation*

*Droplet Size of Uncentrifuged Emulsion*. Droplet size of the uncentrifuged emulsions was determined with an optical microscope (Unilab optical microscope) fitted with X40 objective and a standardized X10 ocular micrometer scale as per the previously reported methods with minor modifications [15]. Geometric mean droplet diameter (MDD) was calculated, and the percent increase in MDD (*X*) of each emulsion formulation after 60 days was determined by the following formula:

(2) $$\begin{array}{c} \%X=100\frac{\left(X_{2}-X_{1}\right)}{X_{1}}, \end{array}$$

where *X*_1_ and *X*_2_ are MDD of the emulsion on day 1 and day 60, respectively. Sampling was done in triplicate, and mean value was calculated.

*Droplet Size of Centrifuged Emulsion*. Emulsion sample of 2 mL was withdrawn into centrifuge tube and subjected to centrifugation at 10000 rpm for 10 min. Emulsions were allowed to stand for 24 h after centrifugation, and MDD was determined. Dispersion ratio (DR) of each emulsion was determined by the following formula [15, 19]:

(3) $$\begin{array}{c} \text{DR}=\frac{{\text{GSD}}_{\text{CE}\;}}{{\text{GSD}}_{\text{UCE}\;}}, \end{array}$$

where GSD_CE_ and GSD_UCE_ are geometric standard deviation of droplet size of centrifuged and uncentrifuged emulsions, respectively.

#### 2.2.3. Basil Oil Cream Formulation 

Experimentally determined rHLB of basil oil was utilized in the development of cream formulation. The composition of basil oil cream is shown in Table 2. Cream formulations were prepared by the previously reported methods [17, 20]. Required blank cream formulations (placebo) were also formulated by the same method.

#### 2.2.4. Antimicrobial Evaluation

Agar diffusion assay was employed for the evaluation of the antimicrobial action of the basil oil and cream formulations against a broad range of microorganisms [21, 22]. For basil oil, 5 mm sterile paper disc (Whatman International Ltd., UK) soaked with 5 *μ*L basil oil was placed in the prelawned plate, and to evaluate the cream formulations (CR4 and CR6), 150 mg was filled into the agar well. A popular marketed antiseptic herbal cream formulation and placebo BCR4 and BCR6 were also employed in the similar manner. All experiments were performed in triplicate, and the zone of growth inhibition (ZGI) was measured as millimeters in diameter.

#### 2.2.5. Skin Irritation Studies

Skin irritation studies were performed for the cream formulations in six healthy New Zealand white rabbits (1500 ± 500 g body weight) as per Federal Hazardous Substances Act (FHSA) guidelines [23, 24]. The experimental protocol has been approved by the Institutional Animal Ethical Committee (Registration no. 400/01/AB/CPCSEA dated 23 September, 2011) of CSIR-CIMAP, Lucknow, India. Cream formulations CR4 and CR6, respective placebo cream (BCR4, BCR6), and marketed antiseptic cream (500 mg) were applied to the previously shaved skin of rabbits on one inch square area. Normal saline (1%) was applied as positive control at opposite side of skin. Observations were made at 4, 24, 48, and 72 h to assess individual erythema and edema using the FHSA recommended Draize scoring criteria. The primary irritation index (PII) was determined using the following formula:

(4) $$\begin{array}{c} \text{PII}=\text{Test}\;\text{Score}-\text{Control}\;\text{Score}. \end{array}$$

#### 2.2.6. Viscosity and Consistency Evaluation

Viscosity of basil oil cream formulations (CR4 and CR6) was determined by DV-II + Pro, viscometer (Brookfield Engineering Laboratories, USA) at 20 rpm, 25 ± 1°C using Spindle 4. Cream formulations (CR4 and CR6) were also evaluated for different consistency (texture) parameters, namely, cream firmness, adhesiveness, cohesiveness, consistency, spreadability, and extrudability by using CT3 Texture Analyzer (Brookfield Engineering Laboratories, USA). Test parameters were selected as per individual test requirement [25, 26]. All the graphs and data were generated using Texture Pro CT V1.3 software, and formulations were evaluated in triplicate.

### 2.3. Statistical Analysis

The Student's *t*-test of significance between MDD of uncentrifuged and centrifuged emulsions and DR of both basil oil and light liquid paraffin emulsions was analyzed using GraphPad InStat Software (Version 5.01, GraphPad Software Inc., USA).

## 3. Results

### 3.1. Creaming Index

Percent CI of basil oil and light liquid paraffin emulsions stored at room temperature is shown in Table 3. On the 5th day the lowest CI, that is, 1.0%, was recorded for basil oil emulsions with HLB 13 (BE9) and HLB 14 (BE10) whereas BE1 (HLB 5) showed the highest CI (3.0%). On the 60th day, all emulsions except BE9 (HLB 13) and BE10 (HLB 14) showed phase separation, and CI was noted to be 9.8% and 10.2%, respectively. Similarly, for light liquid paraffin emulsions, the highest CI was recorded which is shown in Table 3.

### 3.2. Turbidity

**Figure 1:**
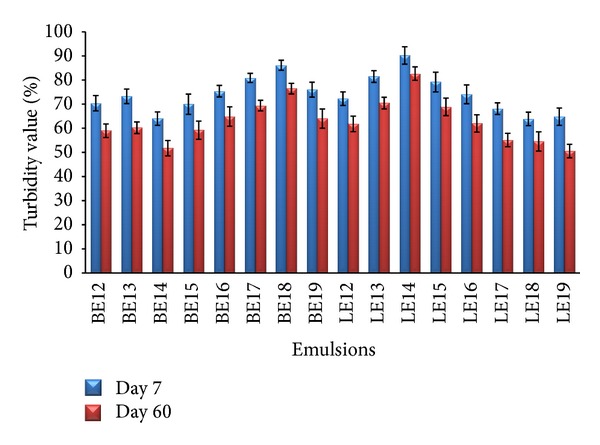
Turbidity of basil oil emulsions and light liquid paraffin emulsions.

Turbidity of basil oil emulsions and light liquid paraffin emulsions is presented in Figure 1. For basil oil emulsions, turbidity was found to be in the range of 86.12 ± 2.1 (BE18; HLB 13.4) to 64.00 ± 2.8 (BE14; HLB 11.8) whereas for light liquid paraffin emulsions, it was found to be between 90.2 ± 3.6 (LE14; HLB 11.8) and 63.9 ± 2.8 (LE18; HLB 13.4) on 7th day. Similarly, turbidity of both oils was recorded on the 60th day of storage.

### 3.3. Droplet Size

**Figure 2:**
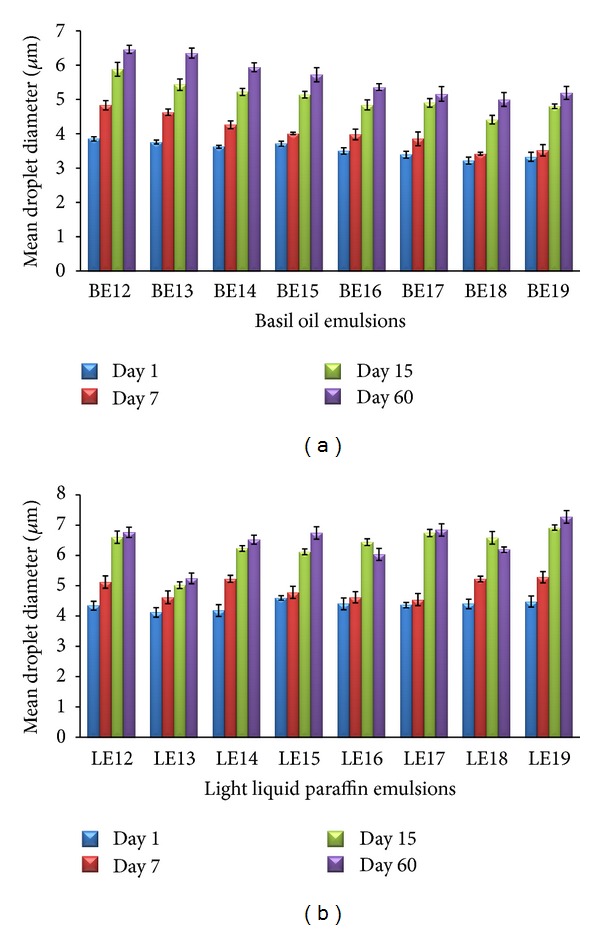
(a) Mean droplet diameter of basil oil emulsion, over 60 days. (b) Mean droplet diameter of light liquid paraffin emulsion, over 60 days.

MDD of uncentrifuged basil oil emulsions and light liquid paraffin emulsions over a period of 60 days is depicted in Figures 2(a) and 2(b), respectively. After one day of formulation, MDD of basil oil emulsions was found to be in the range of 3.22 ± 0.09 *μ*m (BE18) to 3.85 ± 0.12 *μ*m (BE12). The lowest MDD was recorded for BE18 throughout the evaluation. Similarly, for light liquid paraffin emulsions, MDD was in the range of 4.12 ± 0.14 *μ*m (LE13) to 4.60 ± 0.08 *μ*m (LE15) on day one; LE13 (HLB 11.4) showed the lowest MDD for light liquid paraffin emulsions over 60 days.

**Figure 3:**
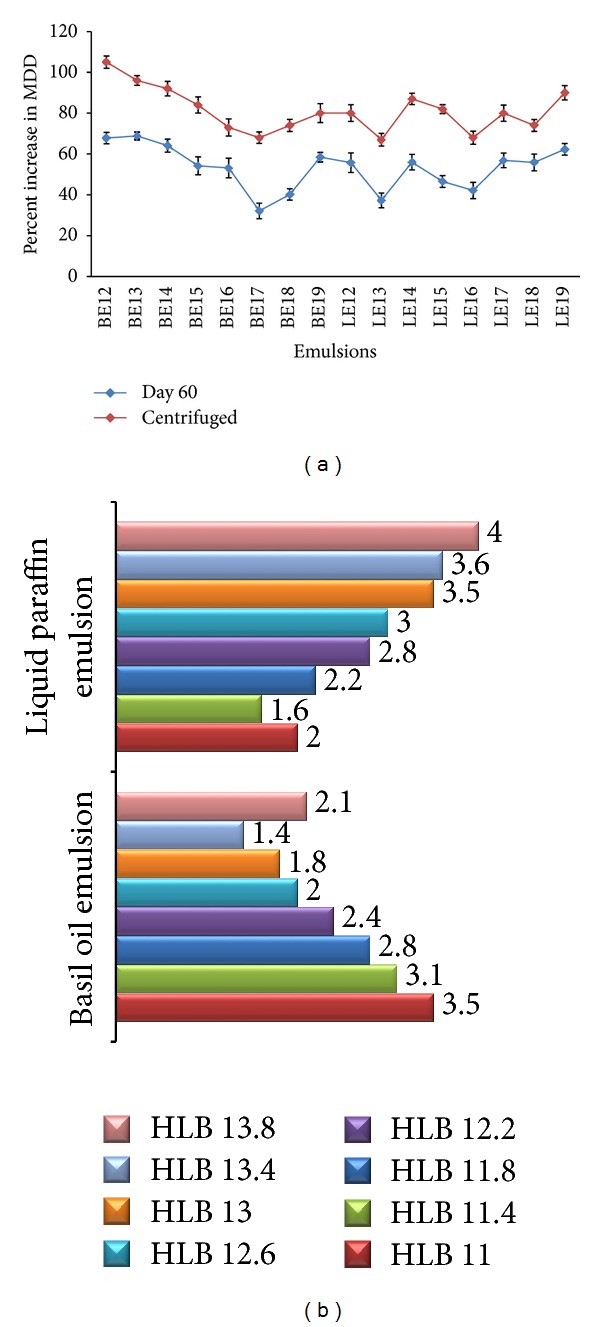
(a) Percent increase in mean droplet diameter of basil oil emulsion and light liquid paraffin emulsion. (b) Dispersion ratio of basil oil emulsion and light liquid paraffin emulsion.

Figure 3(a) shows the percent increase in MDD for both basil oil and light liquid paraffin emulsions upon centrifugation and over 60 days of storage. For basil oil emulsions, the highest and lowest percent increase in MDD was shown by BE13 (HLB 11.4) and BE17 (13.0), that is, 68.88 ± 1.8 and 32.12 ± 3.8%, respectively, whereas light liquid paraffin emulsions exhibited the lowest 37.28 ± 3.6% (LE13; HLB 11.4) and the highest 62.27 ± 2.9% (LE19; HLB13.8) increase in MDD over 60 days of storage. Similar trend was seen upon centrifugation where percent increase in MDD was the highest 105 ± 3% (BE12) and the lowest 68 ± 2.8% (BE17) for basil oil emulsion and the highest 90 ± 3.5% (LE19) and the lowest 67 ± 3.1% (LE13) for light liquid paraffin emulsions.

Figure 3(b) shows the DR of both basil oil and light liquid paraffin emulsions. For basil oil emulsions the lowest DR and the highest DR were 1.4 (BE18) and 3.5 (BE12) whereas for light liquid paraffin emulsions, they were 1.6 (LE13) and 4.0 (LE19), respectively.

### 3.4. rHLB and Cream Formulation

rHLB determined by different evaluation parameters is exhibited in Table 4. rHLB of basil oil and light liquid paraffin was experimentally determined to be 13.36 ± 0.36 and 11.5 ± 0.35, respectively. Utilizing the determined values, the rHLB of cream formulations (Table 2) was calculated to be 12.87. To achieve rHLB 12.87, GMS : Tween 80 in the ratio of 1 : 3.45 and GMS : SLS in the ratio of 3.68 : 1 were taken as two different emulsifier blends. Four cream formulations were formulated with each emulsifier blend, incorporating 2, 4, 6, and 8% of total emulsifier blend concentration. With emulsifier blend 1, 8% (CR4) of total concentration of emulsifier could produce stable cream. Similarly with emulsifier blend 2, 4% (CR6), 6% (CR7), and 8% (CR8) of total concentration of emulsifier could produce stable cream.

### 3.5. Antimicrobial Activity and Skin Irritation Studies

Zone of growth inhibition (ZGI) against tested microbes for basil oil, cream formulation (CR4 and CR6), placebo (BCR4 and BCR6), and marketed herbal antiseptic cream is shown in Table 5. Basil oil was found to be effective against a broad range of microorganisms. Smaller ZGI was recorded for cream CR4 against the tested group of microorganisms compared to basil oil whereas equivalent ZGI was measured for CR6. Marketed antiseptic cream formulation showed the highest ZGI among all the tested microbes.

PII of CR4, CR6, BCR4, BCR6, and marketed herbal antiseptic product was determined to be 0.86, 1.05, 0.38, 0.61, and 0.80, respectively, depicting barely perceptible irritation. 

### 3.6. Viscosity and Consistency

Viscosity of CR4 and CR6 was found to be in the range of 28000–29350 cP. The consistency and other texture parameters, namely, firmness, adhesiveness, extrudability, cohesiveness, and spreadability of the stable cream formulations CR4 and CR6 were found to be steady over the period of three months. Viscosity, consistency, and other texture parameters of basil oil cream formulations CR4 and CR6 are depicted in Table 7.

## 4. Discussion

The basic objective of the present work was to utilize the rHLB value of basil oil in the development of stable cream formulation. The HLB values of basil oil and light liquid paraffin were found to be 13.36 ± 0.36 and 11.5 ± 0.35, respectively [15]. Stable cream formulations CR4 and CR6 were developed with two different emulsifier blends, namely, GMS : Tween 80 (1 : 3.45) and GMS : SLS (3.68 : 1). CR6 showed stronger antimicrobial activity against a broad range of microorganisms than CR4 which may be due to the presence of SLS. Skin irritation studies in rabbits ensured that both of the developed cream formulations were non-irritant in nature.

### 4.1. Creaming, Turbidity, and Droplet Size

Creaming is a natural phenomenon in emulsions, and it may be led by flocculation (reversible aggregation) and/or coalescence (irreversible aggregation) of emulsified droplets, which directly correlate with nature (HLB) and concentration of emulsifiers [27–29]. Creaming Index is a preliminary parameter indicating the stability of emulsions and the nearest range of HLB producing a stable emulsion. Based on the creaming index evaluation, basil oil and light paraffin emulsions in the HLB range of 13-14 and 10–12, respectively, were found to be stable. HLB values of these oils were hypothesized to be within the above mentioned ranges. Therefore, a second series of eight emulsions with HLB values 11.0–13.8 (Table 1) were prepared and subjected to evaluation parameters, namely, particle size and turbidity analysis to reach closer to HLB value.

Stoke's law illustrates that the rate of creaming has a direct relationship with the droplet size, where larger droplets coalesce more rapidly than smaller droplets [30, 31]. It is also apparent that the formation of larger aggregates by coalescence and/or flocculation will accelerate creaming, leading to destabilization of emulsions. Formation of high creaming layer increases the transparency of emulsion whereas emulsions with no/low creaming layer are turbid [17]. Basil oil emulsion BE18; HLB 13.4 and light liquid paraffin emulsion LE14; HLB 11.8 were found to be highly turbid among all emulsions on 7th and 60th day; hence particle size in these emulsions was predicted to be smaller and well dispersed indicating a stable emulsion. Further, all emulsions in the second series were subjected to droplet size analysis.

Degree of dispersion (DR) is reflection of droplet size distribution and previously used in describing skewness/dispersion of a size distribution curve [19]. DR is the ratio of degree of dispersion of both centrifuged and uncentrifuged emulsions. It is obvious that centrifugation at 10000 rpm for 10 min will cause flocculation/coalescence and DR will never be 1, but a closer value indicates a stable emulsion. The Student's *t*-test revealed that there were significant differences between MDD of uncentrifuged and centrifuged emulsions, MDD of centrifuged emulsions and MDD of emulsions on 60th day as well as DR of both basil and light liquid paraffin emulsions.  Table 6 exhibits the calculated and critical *t*-values at *P* = 0.05, *n* = 16. However none of the emulsions cracked after centrifugation or upon storage for 60 days.

Emulsions with the lowest MDD and stumpy percent increase in MDD indicate stable emulsion, which point out the required HLB of the oil.

### 4.2. Antimicrobial and Skin Irritation Studies

Qualitative and quantitative contents of excipients (except emulsifiers) as well as basil oil were the same in both the cream formulation CR4 and CR6; still difference in their antimicrobial activity was observed (Table 5). Further, corresponding placebo formulations BCR4 and BCR6 were assayed, where BCR4 showed activity only against *Staphylococcus epidermidis* (ZGI; 2.3 ± 0.57 mm), and BCR6 was active against all the tested microorganisms (ZGI; 1.0 ± 0.0–3.0 ± 1.0 mm). Based on the findings, it was clear that emulsifier blend 2 (GMS : SLS) in cream CR6 had its own antimicrobial activity; hence, an additive effect was seen. The literature reveals that SLS has its own antimicrobial activity whereas Tweens (nonionic surfactants) reduce antimicrobial activity of phenolic compounds as a result of micellization [6, 32, 33]. Recent findings in the literature reveal that antimicrobial activity of basil oil is not attributable to any specific mechanism as the chemical constituent present in the oil exhibits different precise mode of action [34]. A number of possible mechanisms have been proposed for essential oils, which include degradation of the cell wall, damage to the cytoplasmic membrane, damage to membrane proteins, leakage of cell contents, coagulation of cytoplasm, and depletion of proton motive force [35]. It is therefore likely that the basil oil may act by one or more means to exert its effect. Both the cream formulations CR4 and CR6 were active against microorganisms where CR6 was found to be more promising.

 According to FHSA regulations, a material with PII less than 5.0 is generally not considered as primary irritant to the skin. Basil oil cream formulations exhibited barely perceptible irritation hence considered being non-irritant to skin at the applied dose (500 mg).

### 4.3. Viscosity and Consistency of Cream Formulation

Stoke's law illustrates that viscosity is inversely proportional to sedimentation/creaming; hence high viscosity is desirable in emulsions/creams, but caution must be taken to maintain optimum viscosity which would be acceptable to users. Viscosity of basil oil cream formulations CR4 and CR6 was found to be in the range of 28000–29350 cP. Despite fixed concentration of ingredients, increase in viscosity was observed in CR6, which might be attributed to the high concentration of GMS (Table 2).

Recently, technologies are developed to imitate human perception for semisolid products in terms of consistency, hardness, adhesiveness, cohesiveness, spreadability, and extrudability [25, 36] which are collectively called texture properties of formulation. Hardness of any semisolid formulation is the maximum positive force required to deform the sample with finger. CR6 was comparatively harder than CR4 which is because of the presence of higher concentration of GMS. Adhesiveness, which is defined as the maximum force required for overcoming the attractive force between any surface and sample (cream), was characterized as a measure for stickiness of the sample [25, 37]. Comparatively, CR4 required higher force (1.43 ± 0.05–1.83 ± 0.11 g) than CR6 (0.63 ± 0.05–0.73 ± 0.05 g) in adhesiveness test; hence CR4 was found to be more adhesive. Cohesiveness is another parameter which indicates the strength of internal bonds that is responsible for overall elegance of the cream. Both the creams exhibited similar cohesiveness.

Spreadability of semisolid formulations is one of the important texture parameters which settle on users' preference towards a specific product. Technically, certain amount of work is done to spread the sample (cream) over a surface, and the energy (mJ) required for this purpose defines the spreadability of the formulation [38, 39]. CR4 was comparatively more spreadable than CR6, which is in agreement with the findings that CR6 is harder than CR4. A good formulation with poor extrudability (pattern in which product comes out of container) may not be acceptable as it is an important issue for overall acceptance of formulation by end users [39]. It also indicates any degradation (variation in consistency) or incompatibility of formulation and container. Extrusion of cream was smooth and continuous for both CR4 and CR6 up to three months of study. The values determined for different texture parameters were reproducible over three months and can be utilized as formulation finger print to ensure consistent quality of the developed cream formulation.

## 5. Conclusion

Stable, consistent, non-irritant, enriched antiseptic basil oil cream formulations have been developed utilizing experimentally determined rHLB of basil oil (13.36 ± 0.36). By this novel approach, two different combinations of emulsifiers, that is, GMS : Tween 80 (1 : 3.45) and GMS : SLS (3.68 : 1), could be employed to develop stable antiseptic cream formulation with promising antimicrobial action (net zone of growth inhibition 5.0–11.3 mm against bacteria and 4.3–7.6 mm against fungi).

The novelty of this work is to exploit the lucid science of HLB, which facilitates using a variety of desired emulsifiers/surfactants (as per need or regulatory requirement) to develop a stable antiseptic cream formulation of basil oil. This approach will be of extreme interest for academics R&D as well as pharmaceutical industries involved in the research of biphasic dosage forms.

## Supplementary Material

In the supporting information some literature and our findings (that are not included in the manuscript) are provided for better understanding and recognition of the applied methodologies. As already discussed in the manuscript, we determined the required hydrophilic-lipophilic balance of basil oil, an important physicochemical property. Implementing the determined rHLB value, we further calculated the rHLB of cream formulation and finally developed a stable cream. The formulations were evaluated for antimicrobial activity and skin irritation index. The supporting information includes some representative photographs of antimicrobial evaluation of cream formulations showing zone of growth inhibition (ZGI) against different microorganisms. Details of skin irritation studies are provided which include experimental protocol, scoring criteria, data and calculation of Primary irritation Index.

## Figures and Tables

**Table 1:** Hydrophilic-lipophilic balance spreadsheet of basil oil and light liquid paraffin emulsions.

Basil oil emulsions	HLB	Tween 80 (%)	Span 80 (%)	Light liquid paraffin emulsions
First series emulsions				
BE1	5.0	06.54	93.46	LE1
BE2	6.0	15.88	84.12	LE2
BE3	7.0	25.23	74.77	LE3
BE4	8.0	34.57	65.43	LE4
BE5	9.0	43.92	56.08	LE5
BE6	10.0	53.27	46.73	LE6
BE7	11.0	62.61	37.39	LE7
BE8	12.0	71.96	28.04	LE8
BE9	13.0	81.30	18.70	LE9
BE10	14.0	90.65	09.35	LE10
BE11	15.0	100.00	00.00	LE11

Second series emulsions				
BE12	11.0	62.61	37.39	LE12
BE13	11.4	66.35	33.65	LE13
BE14	11.8	70.09	29.91	LE14
BE15	12.2	73.83	26.17	LE15
BE16	12.6	77.83	22.43	LE16
BE17	13.0	81.30	18.70	LE17
BE18	13.4	85.04	14.96	LE18
BE19	13.8	88.78	11.22	LE19

BE: basil oil emulsion; LE: light liquid paraffin emulsion; HLB: hydrophilic-lipophilic balance.

**Table 2:** Basil oil cream formulation.

Formulations/ingredients (%)	CR1	CR2	CR3	CR4	CR5	CR6	CR7	CR8
Light liquid paraffin	6.00	6.00	6.00	6.00	6.00	6.00	6.00	6.00
White petroleum jelly	2.00	2.00	2.00	2.00	2.00	2.00	2.00	2.00
Basil oil	4.00	4.00	4.00	4.00	4.00	4.00	4.00	4.00
Paraffin wax	2.00	2.00	2.00	2.00	2.00	2.00	2.00	2.00
Cetyl alcohol	3.00	3.00	3.00	3.00	3.00	3.00	3.00	3.00
Stearyl alcohol	3.00	3.00	3.00	3.00	3.00	3.00	3.00	3.00
Water	q.s.	q.s.	q.s.	q.s.	q.s.	q.s.	q.s.	q.s.

	Emulsifier blend 1				Emulsifier blend 2			
	2%	4%	6%	8%	2%	4%	6%	8%

Tween 80	1.55	3.10	4.65	6.20	—	—	—	—
GMS	0.45	0.90	1.35	1.80	1.58	3.15	4.72	6.30
SLS	—	—	—	—	0.42	0.85	1.28	1.70

HLB of cream formulation is 12.87 (calculated).

Emulsifier blend 1: GMS : Tween 80—1 : 3.45.

Emulsifier blend 2: GMS : SLS—3.68 : 1.

GMS: glycerol monostearate; SLS: sodium lauryl sulfate; q.s.: quantity sufficient; CR: cream formulation.

**Table 3:** Creaming index of basil oil and light liquid paraffin emulsions.

Emulsions	HLB	Day 5	Day 10	Day 30	Day 45	Day 60
Basil oil emulsions						
BE1	5.0	3.0	7.6	PS	PS	PS
BE2	6.0	2.8	6.6	PS	PS	PS
BE3	7.0	2.4	6.2	PS	PS	PS
BE4	8.0	2.1	5.9	PS	PS	PS
BE5	9.0	1.8	4.2	PS	PS	PS
BE6	10.0	1.6	4.0	8.2	10.8	PS
BE7	11.0	1.3	3.8	6.6	8.4	PS
BE8	12.0	1.1	3.6	6.4	8.2	PS
BE9	13.0	1.0	3.4	6.0	8.0	9.8
BE10	14.0	1.0	3.2	5.8	7.8	10.2
BE11	15.0	1.1	3.2	6.0	8.0	PS

Light liquid paraffin emulsions						
LE1	5.0	2.8	5.7	PS	PS	PS
LE2	6.0	2.6	5.9	PS	PS	PS
LE3	7.0	2.4	5.8	PS	PS	PS
LE4	8.0	2.0	5.4	PS	PS	PS
LE5	9.0	1.8	4.8	8.9	10.4	PS
LE6	10.0	1.4	3.0	8.6	9.0	10.4
LE7	11.0	1.0	3.0	8.2	8.4	9.2
LE8	12.0	1.0	3.2	8.2	8.8	9.4
LE9	13.0	1.2	3.6	8.4	9.0	PS
LE10	14.0	1.3	3.6	8.6	9.2	PS
LE11	15.0	1.5	3.4	9.0	PS	PS

BE: basil oil emulsion; LE: light liquid paraffin emulsion; PS: phase separation.

**Table 4:** Experimentally determined hydrophilic-lipophilic balance of basil oil and light liquid paraffin.

Evaluation parameters	HLB value producing stable emulsion	
	Basil oil	Light liquid paraffin
Percent CI	13.0, 14.0	11.0, 12.0
MDD	13.4	11.4
Percent increase in MDD	13.0	11.4
DR	13.4	11.4
Turbidity	13.4	11.8

Mean HLB^a^	13.36 ± 0.36	11.5 ± 0.35

HLB: hydrophilic-lipophilic balance; CI: creaming index; MDD: mean droplet diameter; DR: dispersion ratio.

^a^Mean ± SD, *n* = 6.

**Table 5:** Antimicrobial activity of basil oil, developed cream formulations, and marketed herbal antiseptic cream.

Microorganisms	Net zone of inhibition^a^ (mm)					
	Basil oil^b^	CR4^c^	BCR4^c^	CR6^c^	BCR6^c^	Marketed antiseptic cream^c^
Antibacterial activity						
*Staphylococcus aureus *(MTCC 96)	7.3 ± 1.15	5.6 ± 0.57	0	7.6 ± 1.52	1.6 ± 0.57	10.6 ± 1.15
*Staphylococcus epidermidis* (MTCC 435)	13.6 ± 1.52	11.0 ± 1.00	2.3 ± 0.57	10.3 ± 2.0	3.0 ± 1.00	10.0 ± 2.00
*Streptococcus mutans *(MTCC 890)	5.6 ± 1.52	5.6 ± 0.57	0	9.0 ± 1.00	1.6 ± 0.57	7.3 ± 0.57
*Salmonella typhi* (MTCC 733)	6.6 ± 1.15	5.0 ± 1.00	0	7.0 ± 1.00	1.3 ± 0.57	8.6 ± 1.15
*Yersinia enterocolitica* (MTCC 861)	10.0 ± 2.0	6.0 ± 1.00	0	11.3 ± 1.52	1.3 ± 0.57	8.0 ± 2.00
*Klebsiella pneumoniae* (MTCC 109)	4.6 ± 1.15	1.66 ± 0.57	0	7.0 ± 1.00	1.6 ± 0.57	8.6 ± 1.52
*Enterobacter aerogenes *(MTCC 111)	5.0 ± 1.00	1.66 ± 0.57	0	9.0 ± 1.00	1.0 ± 0.00	5.0 ± 1.00

Antifungal activity						
*Candida albicans* (MTCC 1637)	8.6 ± 1.15	4.3 ± 0.57	0	7.0 ± 1.00	1.6 ± 0.57	9.6 ± 1.52
*Aspergillus niger *(MTCC 281)	10.0 ± 2.00	7.3 ± 0.57	0	7.6 ± 1.15	1.0 ± 0.00	7.0 ± 1.00
*Microsporum gypseum *(MTCC 2830)	8.3 ± 1.52	7.0 ± 1.00	0	6.0 ± 1.00	1.0 ± 0.00	6.3 ± 1.15

^a^Mean ± SD, *n* = 3.

^b^Agar disc diffusion method.

^c^Agar well diffusion method.

BCR4 and BCR6; Placebo of CR4 and CR6, respectively.

**Table 6:** The *t*-test of significance between MDD and degree of dispersion of basil oil and light liquid paraffin emulsions.

Test parameters	Basil oil emulsions		Light liquid paraffin emulsions	
	Calculated *t*-value^a^	*P* value	Calculated *t*-value^a^	*P* value
MDD (uncentrifuged versus centrifuged)	9.795	*P* ≤ 0.001	17.97	*P* ≤ 0.001
MDD (centrifuged versus MDD-60th day)	2.520	*P* ≤ 0.01	4.662	*P* ≤ 0.001
Degree of dispersion (uncentrifuged versus centrifuged)	4.228	*P* ≤ 0.001	6.080	*P* ≤ 0.001

^a^ *n* = 16; MDD: mean droplet diameter.

Critical *t*-value = 2.14 at *P* = 0.05.

**Table 7:** Viscosity and texture profile of basil oil cream formulations.

Cream/time interval (days)	Viscosity (cP)	Texture parameters^a^					
		Consistency (mJ)	Firmness (g)	Spreadability (mJ)	Adhesiveness (g)	Cohesiveness	Extrudability (mJ)
CR4							
0	28150 ± 150.00	0.53 ± 0.05	6.30 ± 0.57	1.73 ± 0.05	1.43 ± 0.05	0.97 ± 0.02	27.40 ± 1.21
30	28050 ± 76.37	0.66 ± 0.05	6.60 ± 1.15	1.90 ± 0.10	1.76 ± 0.05	0.96 ± 0.01	28.33 ± 0.50
60	28180 ± 147.42	0.70 ± 0.17	6.60 ± 0.57	1.96 ± 0.11	1.83 ± 0.11	0.95 ± 0.02	28.33 ± 1.30
90	28830 ± 65.06	0.66 ± 0.11	7.60 ± 0.57	2.00 ± 0.20	1.66 ± 0.11	0.98 ± 0.01	28.50 ± 0.51
CR6							
0	28280 ± 100.16	0.73 ± 0.04	7.66 ± 0.57	2.50 ± 0.10	0.73 ± 0.11	0.98 ± 0.01	35.46 ± 0.63
30	28990 ± 132.03	0.76 ± 0.05	8.00 ± 1.00	2.26 ± 0.15	0.73 ± 0.05	0.97 ± 0.02	36.50 ± 0.60
60	28980 ± 130.00	0.76 ± 0.05	8.33 ± 1.15	2.73 ± 0.11	0.63 ± 0.05	0.96 ± 0.01	35.73 ± 1.70
90	29480 ± 140.11	0.80 ± 0.10	8.33 ± 0.57	2.93 ± 0.15	0.73 ± 0.11	0.96 ± 0.02	37.86 ± 1.60

^a^Mean ± SD, *n* = 3.
