# Supplementary material for: Enrichment, Development, and Assessment of Indian Basil Oil Based Antiseptic Cream Formulation Utilizing Hydrophilic-Lipophilic Balance Approach
Source: Biomed Res Int. 2013 Jul 31;2013:410686. doi: 10.1155/2013/410686 (PMC3747473; doi:10.1155/2013/410686)
Supplement: Supplementary file 1 — In the supporting information some literature and our findings (that are not included in the manuscript) are provided for better understanding and recognition of the applied methodologies. As already discussed in the manuscript, we determined the required hydrophilic-lipophilic balance of basil oil, an important physicochemical property. Implementing the determined rHLB value, we further calculated the rHLB of cream formulation and finally developed a stable cream. The formulations were evaluated for antimicrobial activity and skin irritation index. The supporting information includes some representative photographs of antimicrobial evaluation of cream formulations showing zone of growth inhibition (ZGI) against different microorganisms. Details of skin irritation studies are provided which include experimental protocol, scoring criteria, data and calculation of Primary irritation Index. [file 410686.f1.doc]

**Enrichment, development and assessment of Indian basil oil based antiseptic cream formulation utilizing hydrophilic-lipophilic balance approach**

Narayan Prasad Yadav^a^*, Jaya Gopal Meher^a^, Neelam Pandey^a^, Suaib Luqman^b^, Kuldeep SinghYadav^a^, Debabrata Chanda^b^

**SUPPLEMENTARY MATERIALS**

**INTRODUCTION**

The supporting information is provided in order to present the research work in an extensive manner. We have provided some literature information and our findings (that are not included in the manuscript) for better understanding and recognition of the applied methodologies. As already discussed in the manuscript, we determined the required hydrophilic-lipophilic balance of basil oil, an important physicochemical property. Implementing the determined rHLB value, we further calculated the rHLB of cream formulation and finally developed a stable cream. The formulations were evaluated for antimicrobial activity and skin irritation index. The utilization of HLB is a useful approach in development of stable cream formulation. It reduces the formulation attempts and enhances the success rate of formulation development.

The supporting information includes

1. Some representative photographs of antimicrobial evaluation of creams
2. Skin irritation studies
3. **SOME REPRESENTATIVE PHOTOGRAPHS OF ANTIMICROBIAL EVALUATIONS**


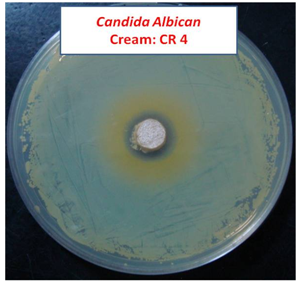

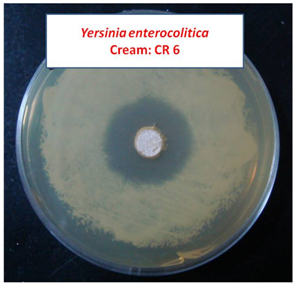

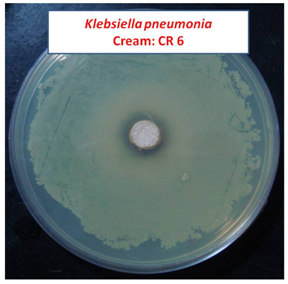

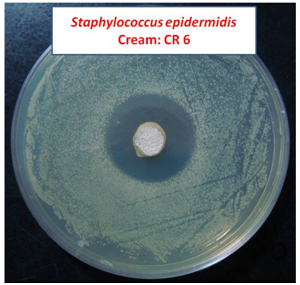

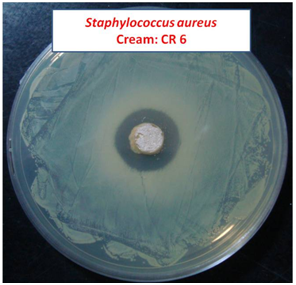

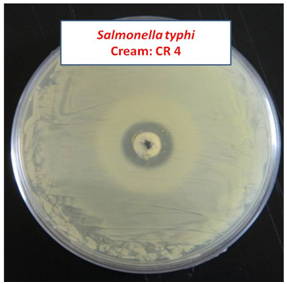


The pictures shown above are the representative photographs of Petri dishes showing the zone of growth inhibition (ZGI) by cream formulations. As discussed in the manuscript, we have taken an marketed herbal antiseptic cream formulation as reference standard for antimicrobial evaluation. The details of the reference standard are given below.

**Cream formulation**: Himalaya antiseptic cream (a popular Indian herbal antiseptic cream)

**Ingredients**: *Aloe vera, Prunus amygdalus, Vitex negundo, Rubia cordifolia, Yashad bhasma (zink calx) and* Sodium tetraborate decahydrate

**Use:** Apply on the affected area 2-3 times daily

**Indication:** Useful in protection against infection in cuts, wound, burns, sores, eruptions, rashes, fungal infections

**Web URL:**  <http://www.himalayahealthcare.com/products/antiseptic_cream.htm>

1. **SKIN IRRITATION STUDIES**

Skin irritation studies were performed as per guidelines of Federal Hazardous Substances Act (FHSA) for the developed Basil oil cream formulations in six healthy New Zealand White rabbits (1500 ± 500 g body weight). The experimental protocol has been approved by the Institutional Animal Ethical Committee (Registration No. 400/01/AB/CPCSEA dated 23^rd^ September 2011) of CSIR-CIMAP, Lucknow, India. The test samples were applied (500 mg) to the previously shaved skin of rats in one inch square area. 1% normal saline was applied as control at opposite side of skin area. The sample applied portions of skin were covered with non reactive sterile tape. After 24 hrs the cream residues were removed and observations were made at 4, 24, 48 and 72 hrs to assess individual erythema and edema using the FHSA recommended Draize scoring criteria. The Primary Irritation Index was determined by the following formula (Aroonrerk & Kamkaen, 2009; Babul, Rehni, Singh, Kao, & Bajaj, 2012).

**Primary Irritation Index (PII) = Test Score – Control Score**

The scores were designated as mentioned in the following table

| Reaction | Gradation | Score |
| --- | --- | --- |
| Erythema | No erythema | 0 |
|  | Very slight erythema (barely observable) | 1 |
|  | Well defined erythema | 2 |
|  | Moderate erythema | 3 |
|  | Severe erythema (prominent redness) | 4 |
| Edema | No edema | 0 |
|  | Very slight edema (barely observable) | 1 |
|  | Well defined edema (edges of test area well defined by definite raising) | 2 |
|  | Moderate edema (raised approximately 1mm) | 3 |
|  | Severe edema (raised more than 1 mm and extending beyond exposure area) | 4 |

**Scoring:**

- Scoring for skin reactions viz. erythema and edema was made by skilled persons on the basis of visual observations.
- The mean of inter hours and inter group of each reaction (erythema and edema) were calculated.
- Finally Grand mean of both reactions (erythema and edema) were determined.
- The scoring details are depicted in the following Tables.

Grand mean is used to determine the Primary Irritation index (PII). Based on the PII the following comments/gradation is assigned to the evaluated samples.

Evaluation of Primary Irritation Index (PII):

| Primary Irritation Index | Comments |
| --- | --- |
| 00 | No irritation |
| 0.04-0.99 | Irritation barely perceptible |
| 1.00-1.99 | Slight irritations |
| 2.00-2.99 | Mild irritation |
| 3.00-5.99 | Moderate irritation |
| 6.00-8.00 | Severe irritation |

**Results**

The results of skin irritation evaluation are depicted in the tables below

**Table 1:** Results of Primary Irritation tests of **control (1% Normal saline)**

| Animal Code† | Score to Skin Reactions | | | | | | | | | | |
| --- | --- | --- | --- | --- | --- | --- | --- | --- | --- | --- | --- |
|  | Erythema | | | | | Edema | | | | | |
|  | Time Duration in Hours | | | | | | | | | | |
|  | 04 | 24 | 48 | 72 | Mean* | | 04 | 24 | 48 | 72 | Mean* |
| 1 (Head) | 0 | 0 | 0 | 0 | 0 | | 0 | 0 | 1 | 0 | 0.25 |
| 2 (Body) | 0 | 0 | 0 | 0 | 0 | | 0 | 0 | 0 | 0 | 0 |
| 3 (Tail) | 0 | 0 | 1 | 0 | 0.25 | | 0 | 1 | 1 | 0 | 0.50 |
| 4 (Head Body) | 0 | 1 | 0 | 0 | 0.25 | | 0 | 0 | 1 | 0 | 0.25 |
| 5 (Body Tail) | 0 | 1 | 0 | 0 | 0.25 | | 0 | 0 | 0 | 0 | 0 |
| 6 (Head Tail) | 0 | 0 | 1 | 1 | 0.50 | | 0 | 0 | 1 | 1 | 0.50 |
| Mean Erythema** | | | | | 0.20 | | Mean Edema** | | | | 0.25 |
| Grand Mean [(Mean Erythema + Mean Edema)/2] | | | | | | | | | | | 0.22 |

†Animals were assigned code by permanent colors, * Inter hrs mean, ** Inter group mean

**Table 2:** Results of Primary Irritation tests of **Basil oil Cream CR4**

| Animal Code† | Score to Skin Reactions | | | | | | | | | | |
| --- | --- | --- | --- | --- | --- | --- | --- | --- | --- | --- | --- |
|  | Erythema | | | | | Edema | | | | | |
|  | Time Duration (hours) | | | | | | | | | | |
|  | 04 | 24 | 48 | 72 | Mean* | | 04 | 24 | 48 | 72 | Mean* |
| 1 (Head) | 0 | 1 | 1 | 2 | 1.00 | | 0 | 1 | 2 | 1 | 1.00 |
| 2 (Body) | 0 | 1 | 1 | 2 | 1.00 | | 0 | 2 | 2 | 1 | 1.25 |
| 3 (Tail) | 0 | 1 | 2 | 1 | 1.00 | | 1 | 2 | 1 | 0 | 1.00 |
| 4 (Head Body) | 0 | 1 | 1 | 2 | 1.00 | | 0 | 2 | 1 | 1 | 1.00 |
| 5 (Body Tail) | 1 | 1 | 2 | 2 | 1.50 | | 0 | 1 | 2 | 1 | 1.00 |
| 6 (Head Tail) | 0 | 2 | 2 | 1 | 1.25 | | 0 | 2 | 1 | 1 | 1.00 |
| Mean Erythema** | | | | | 1.12 | | Mean Edema** | | | | 1.04 |
| Grand Mean [(Mean Erythema + Mean Edema)/2] | | | | | | | | | | | 1.08 |

†Animals were assigned code by permanent colors, * Inter hrs mean, ** Inter group mean

**Table 3:** Results of Primary Irritation tests of **Basil oil Cream CR6**

| Animal Code† | Score to Skin Reactions | | | | | | | | | | |
| --- | --- | --- | --- | --- | --- | --- | --- | --- | --- | --- | --- |
|  | Erythema | | | | | Edema | | | | | |
|  | Time Duration (hours) | | | | | | | | | | |
|  | 04 | 24 | 48 | 72 | Mean* | | 04 | 24 | 48 | 72 | Mean* |
| 1 (Head) | 1 | 1 | 2 | 2 | 1.50 | | 0 | 1 | 2 | 2 | 1.25 |
| 2 (Body) | 0 | 1 | 1 | 2 | 1.00 | | 0 | 2 | 2 | 2 | 1.50 |
| 3 (Tail) | 1 | 1 | 2 | 1 | 1.25 | | 1 | 2 | 1 | 0 | 1.00 |
| 4 (Head Body) | 0 | 1 | 1 | 2 | 1.00 | | 0 | 2 | 1 | 1 | 1.00 |
| 5 (Body Tail) | 1 | 1 | 2 | 2 | 1.50 | | 1 | 1 | 2 | 1 | 1.25 |
| 6 (Head Tail) | 1 | 2 | 2 | 1 | 1.50 | | 0 | 2 | 2 | 2 | 1.50 |
| Mean Erythema** | | | | | 1.29 | | Mean Edema** | | | | 1.25 |
| Grand Mean [(Mean Erythema + Mean Edema)/2] | | | | | | | | | | | 1.27 |

†Animals were assigned code by permanent colors, * Inter hrs mean, ** Inter group mean

**Table 4:** Results of Primary Irritation tests of **Placebo Cream BCR4**

| Animal Code† | Score to Skin Reactions | | | | | | | | | | |
| --- | --- | --- | --- | --- | --- | --- | --- | --- | --- | --- | --- |
|  | Erythema | | | | | Edema | | | | | |
|  | Time Duration (hours) | | | | | | | | | | |
|  | 04 | 24 | 48 | 72 | Mean* | | 04 | 24 | 48 | 72 | Mean* |
| 1 (Head) | 0 | 1 | 1 | 1 | 0.75 | | 0 | 1 | 1 | 1 | 0.75 |
| 2 (Body) | 0 | 1 | 0 | 1 | 0.50 | | 0 | 0 | 1 | 1 | 0.50 |
| 3 (Tail) | 0 | 1 | 1 | 1 | 0.75 | | 0 | 1 | 1 | 0 | 0.50 |
| 4 (Head Body) | 0 | 1 | 0 | 1 | 0.50 | | 0 | 1 | 0 | 1 | 0.50 |
| 5 (Body Tail) | 0 | 1 | 1 | 1 | 0.75 | | 0 | 0 | 1 | 1 | 0.50 |
| 6 (Head Tail) | 0 | 1 | 1 | 1 | 0.75 | | 0 | 0 | 1 | 1 | 0.50 |
| Mean Erythema** | | | | | 0.66 | | Mean Edema** | | | | 0.54 |
| Grand Mean [(Mean Erythema + Mean Edema)/2] | | | | | | | | | | | 0.60 |

†Animals were assigned code by permanent colors, * Inter hrs mean, ** Inter group mean

**Table 5:** Results of Primary Irritation tests of **Placebo Cream BCR6**

| Animal Code† | Score to Skin Reactions | | | | | | | | | | |
| --- | --- | --- | --- | --- | --- | --- | --- | --- | --- | --- | --- |
|  | Erythema | | | | | Edema | | | | | |
|  | Time Duration (hours) | | | | | | | | | | |
|  | 04 | 24 | 48 | 72 | Mean* | | 04 | 24 | 48 | 72 | Mean* |
| 1 (Head) | 1 | 1 | 1 | 1 | 1.00 | | 0 | 1 | 1 | 1 | 0.75 |
| 2 (Body) | 0 | 1 | 1 | 1 | 0.75 | | 0 | 0 | 1 | 1 | 0.5 |
| 3 (Tail) | 1 | 1 | 1 | 1 | 1.00 | | 1 | 1 | 1 | 0 | 0.75 |
| 4 (Head Body) | 0 | 1 | 1 | 1 | 0.75 | | 0 | 1 | 1 | 1 | 0.75 |
| 5 (Body Tail) | 1 | 1 | 1 | 1 | 1.00 | | 1 | 1 | 1 | 1 | 1.00 |
| 6 (Head Tail) | 1 | 1 | 1 | 1 | 1.00 | | 0 | 1 | 1 | 1 | 0.75 |
| Mean Erythema** | | | | | 0.91 | | Mean Edema** | | | | 0.75 |
| Grand Mean [(Mean Erythema + Mean Edema)/2] | | | | | | | | | | | 0.83 |

†Animals were assigned code by permanent colors, * Inter hrs mean, ** Inter group mean

**Table:** Results of Primary Irritation tests of **marketed herbal antiseptic cream**

| Animal Code† | Score to Skin Reactions | | | | | | | | | | |
| --- | --- | --- | --- | --- | --- | --- | --- | --- | --- | --- | --- |
|  | Erythema | | | | | Edema | | | | | |
|  | Time Duration (hours) | | | | | | | | | | |
|  | 04 | 24 | 48 | 72 | Mean* | | 04 | 24 | 48 | 72 | Mean* |
| 1 (Head) | 0 | 1 | 1 | 2 | 1.00 | | 0 | 1 | 2 | 1 | 1.00 |
| 2 (Body) | 0 | 1 | 1 | 2 | 1.00 | | 0 | 2 | 2 | 1 | 1.25 |
| 3 (Tail) | 0 | 1 | 2 | 1 | 1.00 | | 1 | 2 | 1 | 0 | 1.00 |
| 4 (Head Body) | 0 | 1 | 1 | 1 | 0.75 | | 0 | 2 | 1 | 1 | 1.00 |
| 5 (Body Tail) | 1 | 1 | 2 | 1 | 1.25 | | 0 | 1 | 2 | 1 | 1.00 |
| 6 (Head Tail) | 0 | 2 | 1 | 1 | 1.00 | | 0 | 2 | 1 | 1 | 1.00 |
| Mean Erythema** | | | | | 1.00 | | Mean Edema** | | | | 1.04 |
| Grand Mean [(Mean Erythema + Mean Edema)/2] | | | | | | | | | | | 1.02 |

† Animals were assigned code by permanent colors, * Inter hrs mean, ** Inter group mean

**Calculation of Primary Irritation Index:**

**Basil oil Cream CR4**

Primary Irritation Index (PII) = Test PII – Control PII

= 1.08 – 0.22 = **0.86**

**Basil oil Cream CR6**

Primary Irritation Index (PII) = Test PII – Control PII

= 1.27 – 0.22 = **1.05**

**Placebo Cream BCR4**

Primary Irritation Index (PII) = Test PII – Control PII

= 0.60 – 0.22 = **0.38**

**Placebo Cream BCR6**

Primary Irritation Index (PII) = Test PII – Control PII

= 0.83 – 0.22 = **0.61**

**Marketed herbal antiseptic cream**

Primary Irritation Index (PII) = Test PII – Control PII

= 1.02 – 0.22 = **0.80**

**Conclusion:**

According to FHSA (Federal Hazardous Substances Act) regulations, a material with a PII of less than 5.00 is generally not considered a primary irritant to the skin. PII of Basil oil cream formulation CR4 was determined to be 0.86 (Irritation barely perceptible), whereas for CR6 it was 1.05 (Irritation barely perceptible) and hence, these creams are considered to be non irritant to skin.

**REFERENCES**

Aroonrerk, N., & Kamkaen, N. (2009). Anti-inflammatory activity of Quercus infectoria, Glycyrrhiza uralensis, Kaempferia galanga and Coptis chinensis, the main components of Thai herbal remedies for aphthous ulcer. Journal of Health Research, 23(1), 17-22.

Babul, N., Rehni, A., Singh, A., Kao, H., & Bajaj, A. (2012). Skin irritation potential of topical mepivacaine gel and cream dosage forms. The Journal of Pain, 13(4, Supplement), S87.
